# Probing the mechanical stability of bridged DNA-H-NS protein complexes by single-molecule AFM pulling

**DOI:** 10.1038/s41598-017-15477-4

**Published:** 2017-11-10

**Authors:** Yan Liang, Ramon A. van der Valk, Remus T. Dame, Wouter H. Roos, Gijs J. L. Wuite

**Affiliations:** 1 Function Laboratory for Marine Fisheries Science and Food Production Processes, Qingdao National Laboratory for Marine Science and Technology; Yellow Sea Fisheries Research Institute, Chinese Academy of Fishery Sciences, Qingdao, China; 2 Department of Physics and Astronomy and LaserLab, Vrije Universiteit Amsterdam, Amsterdam, The Netherlands; 3 Leiden Institute of Chemistry and Cell Observatory, Leiden University, Leiden, The Netherlands; 4 Moleculaire Biofysica, Zernike instituut, Rijksuniversiteit Groningen, Groningen, The Netherlands

## Abstract

Atomic force microscopy (AFM) has proven to be a powerful tool for the study of DNA-protein interactions due to its ability to image single molecules at the nanoscale. However, the use of AFM in force spectroscopy to study DNA-protein interactions has been limited. Here we developed a high throughput, AFM based, pulling assay to measure the strength and kinetics of protein bridging of DNA molecules. As a model system, we investigated the interactions between DNA and the Histone-like Nucleoid-Structuring protein (H-NS). We confirmed that H-NS both changes DNA rigidity and forms bridges between DNA molecules. This straightforward methodology provides a high-throughput approach with single-molecule resolution which is widely applicable to study cross-substrate interactions such as DNA-bridging proteins.

## Introduction

Single molecule manipulation and force spectroscopy approaches such as optical tweezers, magnetic tweezers, acoustic force spectroscopy (AFS) and atomic force microscopy (AFM) have emerged as powerful biophysical techniques^1–5^. Through their ability to precisely and accurately measure displacements and forces, these techniques offer researchers the opportunity to directly study the interactions of biological systems. Following its primary use in obtaining high-resolution topographical images, AFM has been widely used to determine physical properties of specimens and for measuring a variety of interaction forces. The strength of AFM approaches lies in the ability to perform high-throughput *in-liquid* measurements on individual molecules or particles, thereby generating statistically relevant data in only a few measurement cycles^6^. In single molecule micromechanical AFM experiments, fundamental intramolecular and intermolecular interactions, such as measurements of strength of molecular bonds between ligands and receptors, DNA-protein complexes and unfolding energy landscapes of proteins have been investigated^7–11^.

H-NS is an example of a protein well studied by a myriad of single-molecule techniques. AFM imaging has shown that H-NS forms and stabilizes loops in DNA via intramolecular DNA bridges^12,13^. The ability of H-NS to bridge DNA was later confirmed in optical tweezers experiments^14^. The functional repertoire of H-NS was expanded to include a non-bridging mode in which the protein binds along DNA to form stiff filaments^15^. Mg^2+^-ions have been proposed to play an important role in driving a transition between the two types of H-NS/DNA complexes^16^. This switch between two types of complexes may be connected to a function of H-NS as environmental sensor, responding to environmental cues^17^. The ability to rapidly, and with high-throughput, determine the influence of environmental factors on the action of proteins that bridge DNA such as H-NS would help to further expand our knowledge of this important class of proteins^18^.

In this study, we demonstrate a high-throughput AFM pulling assay to measure the rupture kinetics of DNA-H-NS-DNA protein complexes. By acquiring force-distance curves, we gain information on the forces associated with H-NS induced changes in DNA rigidity and the formation of H-NS bridges between DNA molecules. This simple approach allows measurements on DNA-protein interaction with single-molecule resolution and is widely applicable to other - especially divalent - DNA-binding proteins and receptor-ligand interactions.

## Results

**Figure 1:**
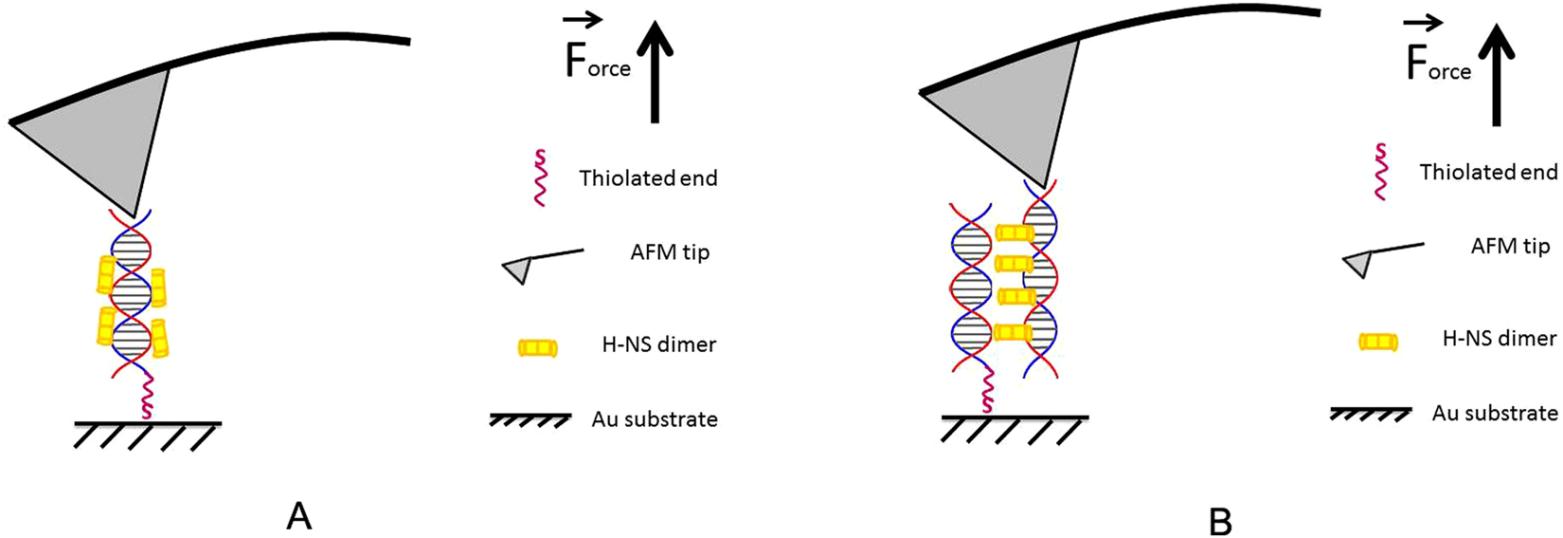
Schematic view of protein-DNA and DNA-protein-DNA complex pulling experiments. The DNA molecules are immobilized on the surface via an Au-S bond between a thiolated DNA extremity and the Au-coated surface. An untreated SiN AFM tip is used to capture the other extremity of the DNA-protein complexes. (**A**) Pulling on an H-NS-DNA complex. (**B**) Pulling on a bridged DNA-H-NS-DNA complex.

In order to investigate the interaction between DNA and H-NS we use DNA with a thiol at one of its ends. These DNA molecules connect covalently to the gold surface of our fluidic chamber. Next, we introduce H-NS or H-NS and free DNA into the sample chamber to let it react with the tethered DNA. Note that we exclude interaction of *E. coli* H-NS with the gold surface via a cysteine residue at position 21 by using a functionally unaltered^19^ H-NS_C21S_ mutant. We will refer to this mutant in the text as H-NS. The label-free ends of the DNA molecules can anchor via non-specific interaction to the AFM tip by pushing the tip onto the surface (Fig. 1). When a connection is established a stretching curve is generated. Typically, we move the tip a distance twice the DNA contour length away from the surface to ensure that the DNA molecules break off the tip after this single stretching curve. Figure 1 shows the schematic view of the DNA-H-NS (Fig. 1A) or DNA-H-NS-DNA complex (Fig. 1B) stretched by an AFM tip.

**Figure 2:**
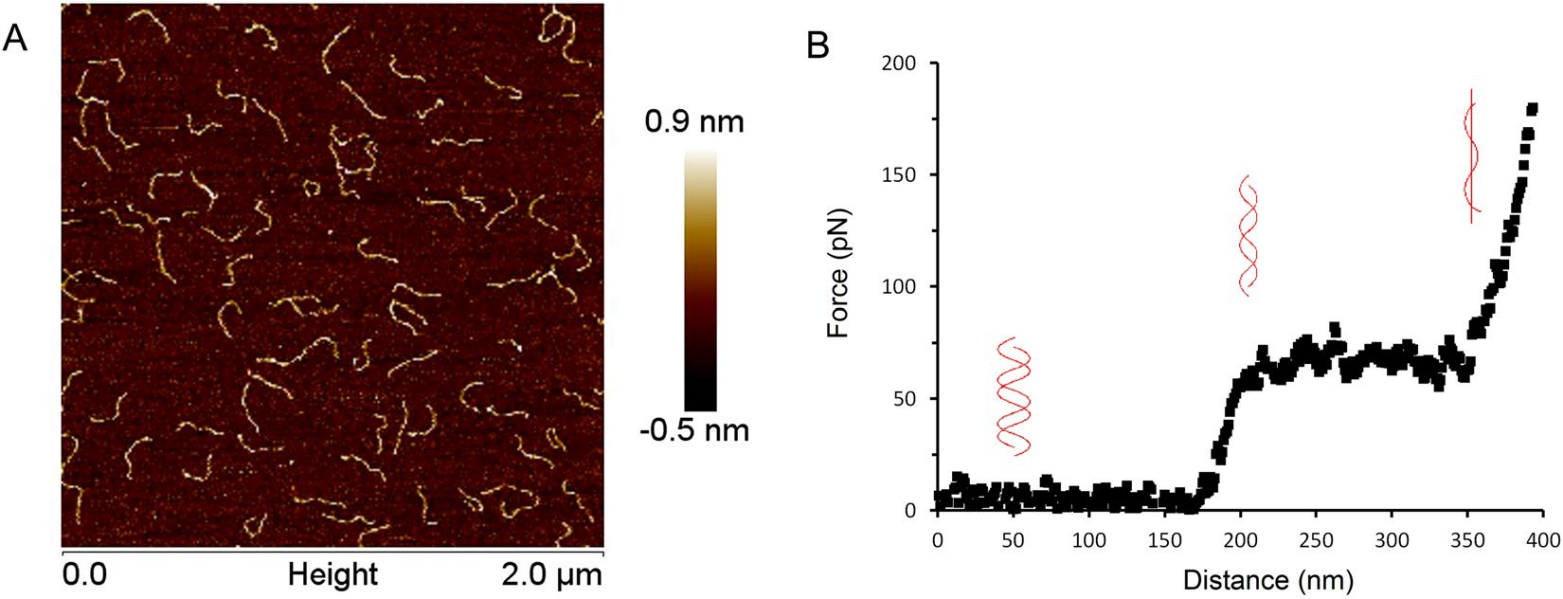
Imaging and pulling of DNA molecules by AFM. (**A**) Imaging of DNA molecules with concentration of 5.42 ng/µl on mica in air. (**B**) Typical FD curve obtained by pulling a single dsDNA by AFM in liquid. Insets illustrate associated structural transitions. Pulling rate at 200 nm/sec.

In order to obtain optimum conditions for catching DNA molecules between the AFM tip and the surface, we first established a suitable DNA concentration on the surface. We determined the tethered DNA concentration on the mica surface by AFM imaging. This imaging had to be done in air because in liquid conditions the DNA tethers move around and thus can’t be imaged. Figure 2A shows a typical topographical image of bare tethered DNA molecules. These DNA molecules have contour length of 220 nm ± 10 nm (N = 23), close to the expected contour length of 232 nm. A suitable DNA concentration for our experiments was defined based on estimates of the surface binding density of deposited DNA. With a 50-fold dilution, resulting in a final concentration of 5.42 ng/µl, tethered DNA molecules no longer overlapped when deposited on the surface which ensures that during pulling experiments it is less likely that the AFM tip can interact with more than one DNA molecule. In all following experiments the DNA samples were diluted 50-fold or more to concentrations of 5.42 ng/µl, 3.5 ng/µl or 1.46 ng/µl.

**Figure 3:**
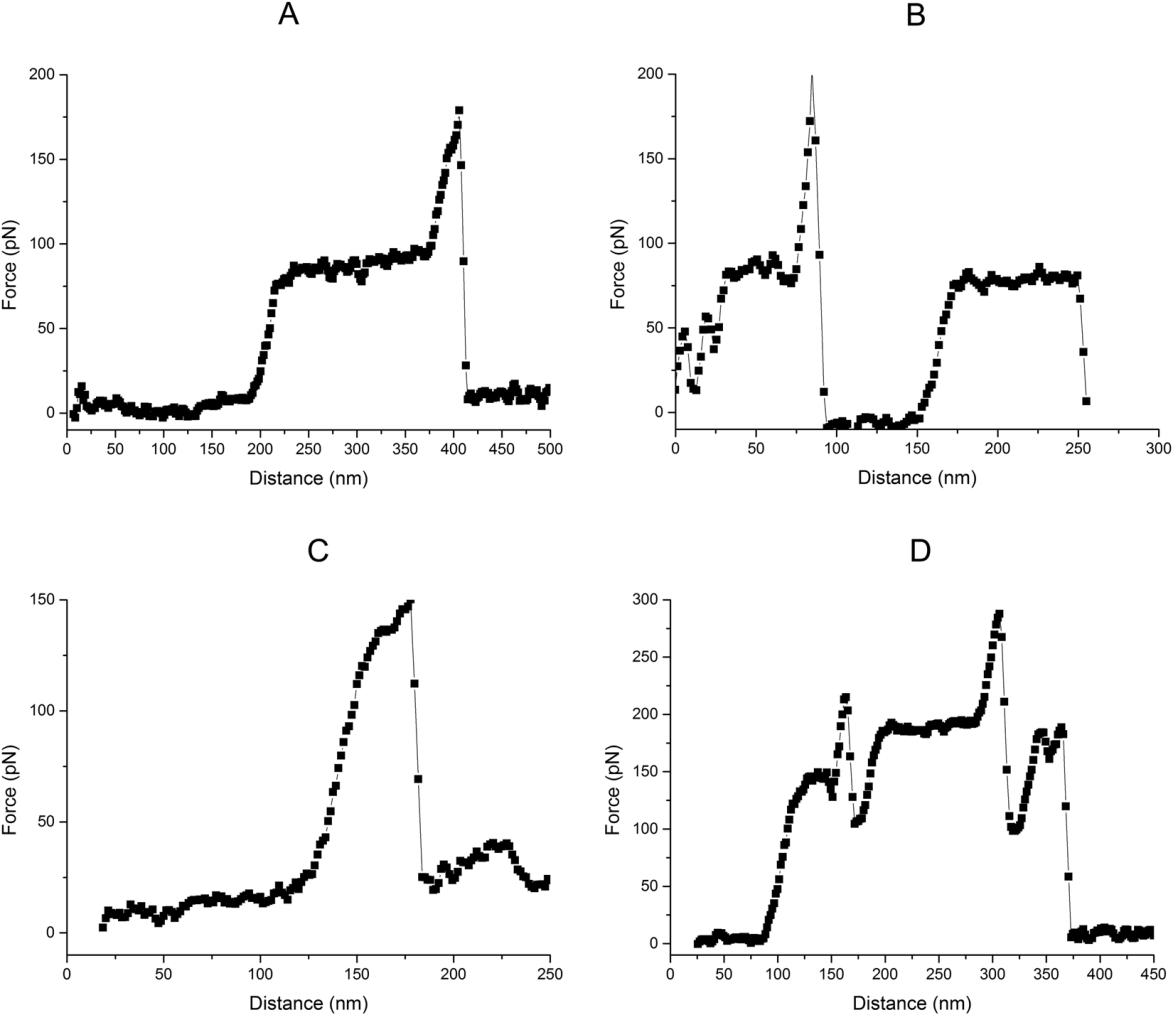
Typical FD curves corresponding to different types of complexes. (**A**) “Single DNA”, FD curve from pulling single DNA; (**B**) “Repeated single DNAs”, FD curve from the event that is either bound to two sites of one DNA or to two DNAs with different lengths due to the non-specific nature of the interaction with the tip; (**C**) “Dual DNA”, FD curve from pulling two DNAs at the same time; (**D**) “Multiple DNAs”, FD curve with multiple different DNA-tip interactions.

Next, pulling experiments were carried out in liquid on bare DNA and the resulting Force-Distance (FD) curves were recorded (Fig. 2B). Bare single dsDNA molecules yield FD curves with the expected characteristic features such as overstretching at around 65 pN^6,20,21^. At high force (>200 pN at a pulling rate of 200 nm/sec) the sample detaches from the tip. The FD curves of each measurement were classified according to their characteristics. Figure 3 shows typical FD curves to illustrate the criteria used for grouping those curves. The groups are defined as: (a) “Single DNA”: one overstretching plateau, overstretching force between 60 to 90 pN, the transition into overstretching displays a rounded corner and no significant force jumps are seen during overstretching. This class represents clean overstretching curves of bare DNA with all expected features; (b) “Repeated single DNAs”: as previous class but with two overstretching plateaus visible. In this case the tip is either bound to two sites of one DNA or to two DNAs with different lengths due to the non-specific nature of the interaction with the tip; (c) “Dual DNA”: one overstretching plateau, with an overstretching force that is larger than 100 pN suggestive of two DNA molecules being pulled simultaneously with the AFM tip; (d) “Multiple DNAs”: displaying a mixture of interactions and are not suitable for further analysis.

**Figure 4:**
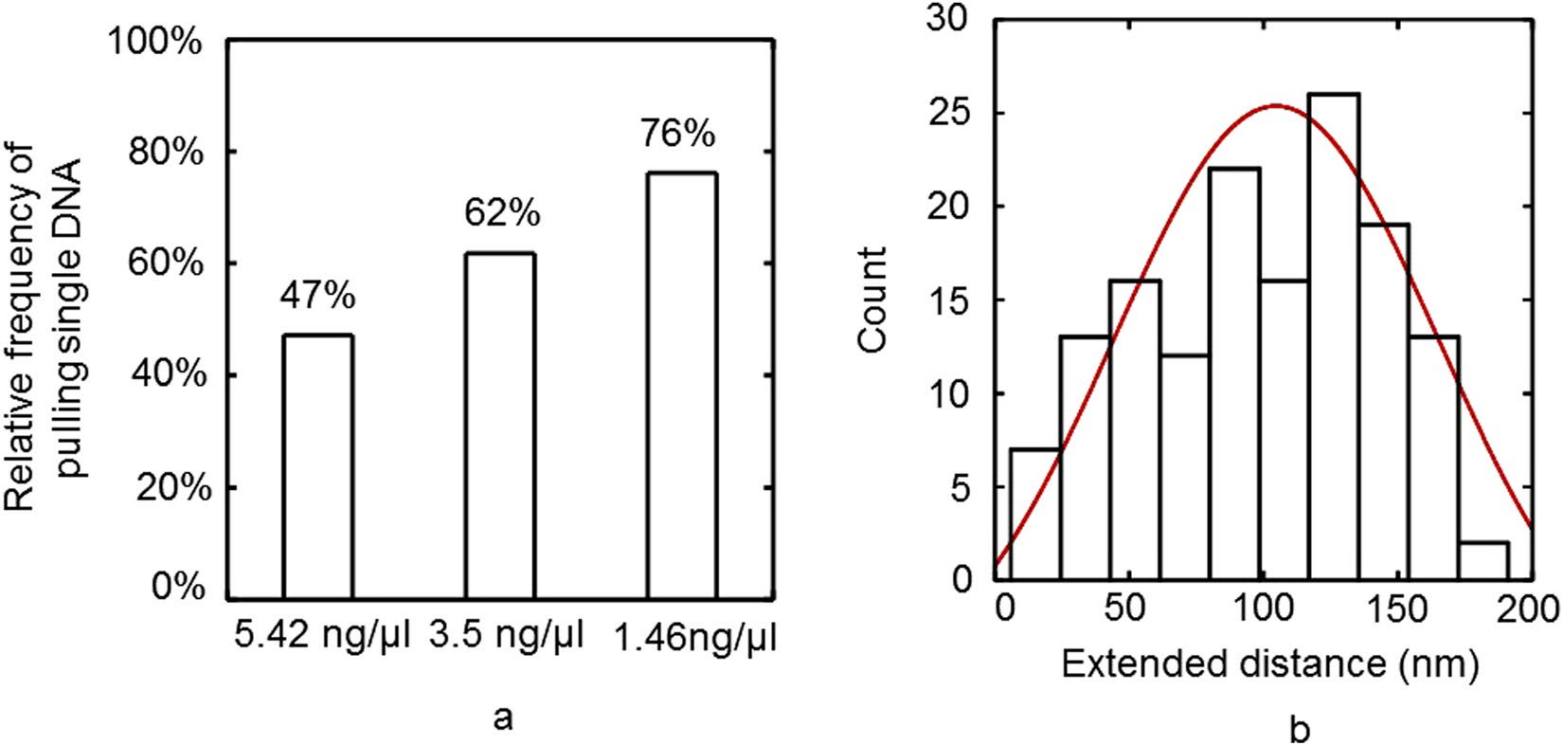
Analysis of pulling a single DNA molecule. (**a**) Relative frequency at which a single DNA molecule is pulled in relation to the DNA concentration (N = 49 for 5.42 ng/µl, N = 89 for 3.5 ng/µl and N = 33 for 1.46ng/µl). (**b**) - Distribution of contour lengths of the DNA molecules pulled by AFM (N = 146). The values are fitted to a Gaussian (red) yielding an average DNA contour length of 105 ± 3.2 nm.

Finding an optimum concentration of tethered DNA molecules on the surface is important to maximize the frequency that a single DNA molecule is picked up in a single tip approach and retract measurement. Thus, pulling events at 3 different DNA concentrations (5.42 ng/µl, 3.5 ng/µl, 1.46 ng/µl) were compared and classified according to the criteria illustrated in Fig. 3. These experiments show an increasing probability (from 47% to 76%) of pulling single DNA molecules (“Single DNA” events) when we decrease the DNA concentration (Fig. 4a). Note that the pulling efficiency, i.e. the number of actual pulling events, also dropped with the DNA concentration. At the lowest tested concentration ~10% of all approach and retract cycles showed an interaction with DNA. From the “Single DNA” FD curves we extracted the contour length i.e. the length of the DNA between the tip and surface. The average contour length we found was 105 ± 3 nm (N = 150; SEM), roughly half the contour length of the used DNA fragment (232 nm, assuming B-DNA) indicating that the DNA molecules are, as expected, randomly attached to the tip (Fig. 4b). Based on this data we conclude that the surfaces prepared with 1.46 ng/µl DNA are suitable for measurements on more complex DNA-protein interactions, because the majority of pulling events yield clean FD curves on single DNA tethers.

**Figure 5:**
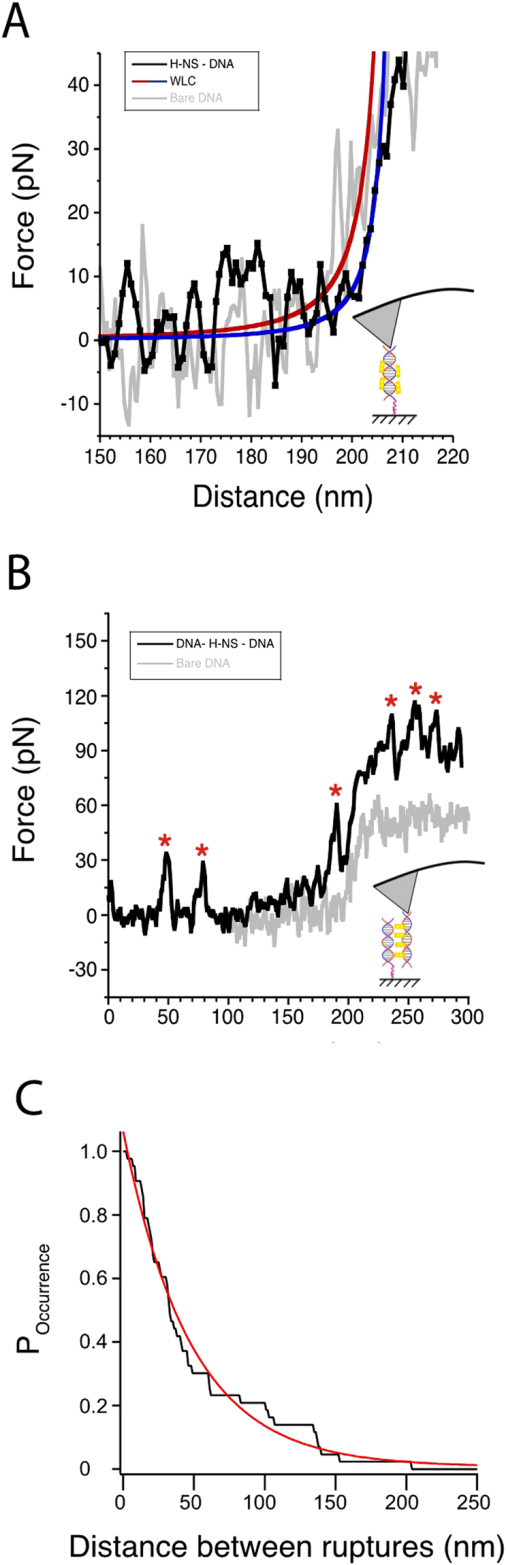
Typical curves of pulling DNA-H-NS (-DNA) complexes. (**A**) In black, a FD curve of a DNA molecule covered with H-NS. For comparison, a bare DNA molecule is shown in grey. The curves are fitted to a WLC curve (with a persistence length of 23 nm ± 2 nm for bare DNA and 49 nm ± 3 nm for the H-NS-DNA filaments) in order to assess quantify the stiffness of the molecule. (**B**) A DNA-H-NS-DNA is shown in black. Asterisks indicate breaking events we observe when we stretch such complex. We did not observe a preferred length of released DNA from such rupture events. (**C**) The distances between rupture events (as observed in Fig. 5B). The probability of finding a specific distance between ruptures (P_occurrence_), as a function of the rupture distance. The curve was fit with an exponential decay function with a decay rate of 48 ± 1 nm.

With optimal conditions established we started to compare FD curves of bare DNA with those of DNA-H-NS and DNA-H-NS-DNA complexes. When we introduce H-NS (2 μM) or H-NS (1 μM) & DNA fragments (2.0 ng/μl), we observed H-NS-DNA filaments (“Stiff DNA”) (Fig. 5A), and “Bridged DNA” complexes (DNA molecules bridged by H-NS) (Fig. 5B). Previous single-molecule measurements with optical tweezers and magnetic tweezers reported that H-NS-DNA filaments are stiffer compared to DNA (i.e. displaying a persistence length of 80 nm, rather than the 50 nm of bare DNA)^14^^,^^15^. Although at a much lower resolution, in our assay we also observe a clear increase in the persistence length of the DNA upon binding of H-NS (compare Fig. 5A). The “Bridged DNA” DNA-H-NS-DNA curves, on the other hand, show breaking point events (sharp decreases in force) during stretching (Fig. 5B). The fact that we observe multiple breaks in a single pulling experiment is probably caused by the entanglement of two DNA molecules each ~5 persistence length long, because of their length multiple bridges can be formed between the DNA molecules. Moreover, entanglements of multiple DNA molecule could also be possible given that we introduced DNA in solution. The rupture events are independent/uncorrelated events that follow an exponential decay (Fig. 5C). The break events we observe appear similar to ruptured intramolecular bridges as seen in optical tweezers measurements^14^.

**Figure 6:**
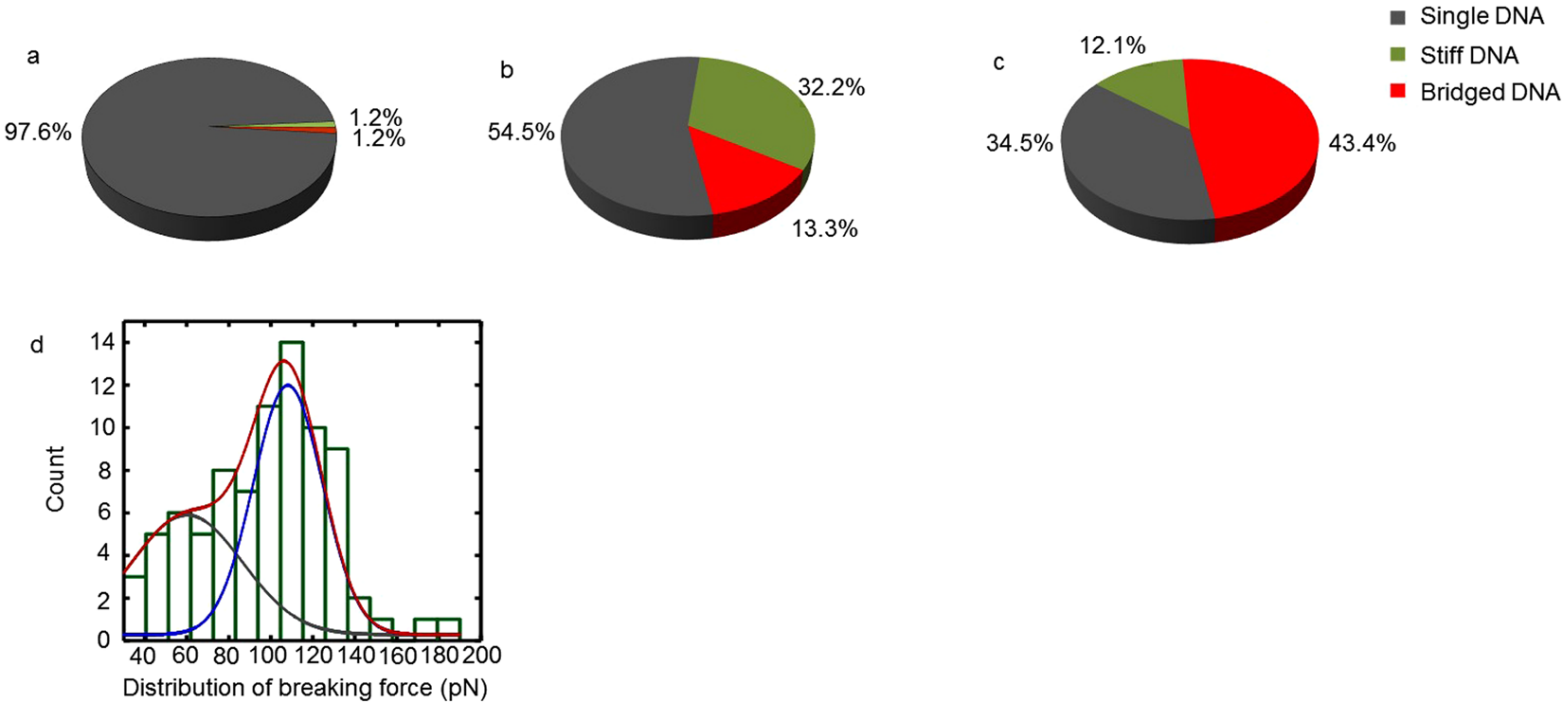
Comparison of pulling events in each measurement. (**a**) The distribution of different types of complexes obtained in buffer with only DNA tethered to the surface (N = 86); (**b**) the distribution of different types of complexes obtained in solution containing H-NS (N = 111); (**c**) the distribution of different types of complexes obtained in solution with both DNA and H-NS (N = 76). (**d**) the distribution of breaking force of protein-bridges between DNA fragments. The values are fitted to a dual peak Gaussian distribution, which give two peaks: one at ~60 pN and another at ~110 pN.

To robustly test the influence of H-NS on DNA, we performed many pulling experiments in three different conditions: (i) only DNA tethers; (ii) DNA tethers with H-NS in solution and (iii) DNA tethers and H-NS & free DNA in solution. The FD curves obtained in these experiments were classified in the three types of behaviour as defined before, “Single DNA”, “Stiff DNA” and “Bridged DNA” (Fig. 6). Figure 6a confirms that without protein we mostly observe FD curves of single tethered DNA molecules. Figure 6b shows that, in the presence of H-NS, around a third of the DNA molecules are stiffened (~32%), a typical characteristic of H-NS binding to DNA (see refs^14,15^). Some FD curves (~13%) show “Bridged DNA” behavior under these conditions. In contrast, experiments in which we introduce both H-NS as well as DNA fragments, the quantity of “Bridged DNA” events increased to 43%, while the number of “Stiff DNA” events decreased to 12% (Fig. 6c). We also determined the distribution of rupture forces observed in “Bridged DNA” FD curves (Fig. 6d). The values obtained are fitted to a double Gaussian distribution resulting in an average force of 60 ± 20 pN and 110 ± 10 pN (N = 69). Based on this data we believe that these rupture events are the result of disrupting DNA-H-NS-DNA complexes because the breaks are mostly seen when free DNA and H-NS are present and because the observed forces are consistent with published rupture forces of H-NS bridges^13,14,22^. The double distribution is likely a reflection of rupturing the DNA-H-NS-DNA interactions in either a sheering or an unzipping geometry^14^ with the later geometry requiring a significant lower force to disrupt.

## Discussion

This study describes a promising application of AFM to measure DNA-H-NS-DNA complexes. H-NS has been proposed to be involved in structuring the nucleoid and in gene regulation. Two different modes of binding have been reported: a stiffening mode and a bridging mode^13,15,22,23^. In the stiffening mode, H-NS multimerizes along DNA, forming a complex of higher bending rigidity. In the bridging mode, H-NS induces bridging of two DNA segments. It has been proposed that a transition between these two binding modes of H-NS occurs, which is driven by changes in the concentration of divalent cations, such as Mg^2+^ or Ca^2+^ ^16,24^. Using an AFM pulling assay we confirmed that H-NS both changes DNA rigidity and forms bridges between DNA molecules in the presence of Mg^2+^. This result is in accordance with the observation that H-NS binding to DNA occurs either in a bridging mode or a stiffening mode, and that these two modes are switchable upon changes in environmental conditions^16,24^. But note that recent studies suggest that the DNA bridging mode is likely physiologically more relevant^24,25^. This novel assay provides a high-throughput tool to probe rupture kinetics of the DNA-H-NS-DNA bridges under diverse conditions.

In our AFM method we have quantified the rupture force of H-NS bridges and observe that a large fraction of the H-NS bridges are strong enough to survive into the overstretching regime (>65 pN). Yet, it is important to consider that the resilience of H-NS DNA interactions to force, depends strongly on the speed at which the force is applied, this was clearly shown by Dame *et al*.^14^, where they used optical tweezers to study the strength of H-NS DNA bridges. With the AFM we pull considerably faster (400 nm/sec) than with the optical tweezers (22 nm/sec) hence we see rupture at these high forces.

In conclusion, we have described an AFM-based technique to measure interaction forces of DNA-protein complexes in high throughput. This method is optimized to probe individual DNA-protein complexes in a population and to sample many complexes by fast scanning of the AFM tip over the surface. In this way, we can perform approach/retract cycles for hundreds of positions which results in an overall distribution of the interaction forces between DNA and its binding protein as characterized by their F-D curves. This technique is very suitable for high throughput analysis albeit at a lower resolution than other assays such as optical tweezers which typically have a lower throughput. The method for sample preparation is relatively simple and quick compared to optical tweezers. The described approach promises a wide applicability, not only to study DNA-H-NS interactions, but also for studies of binding strengths and dynamics of other DNA-binding and bridging proteins.

## Methods

### Preparation of DNA substrate

A double-stranded DNA substrate of 685 basepairs in length, with 32% GC content, was generated by PCR as described previously^26^. For gold surface attachment a 5′ thiol-labelled variant of one of the primers was used (Eurogentec, The Netherlands).

### Construction of H-NS_C21S_ expression vector

A PCR fragment containing the *hns* gene mutated to encode serine instead of cysteine at position 21 was generated by PCR based site-directed mutagenesis on an *E. coli* genomic template. The vector pRD41 for expression of H-NS_C21S_ was constructed by inserting this fragment into pET3His using NdeI and XhoI restriction sites. By incorporating a stop codon directly upstream of the XhoI restriction site the encoded protein does not contain a C-terminal His-tag.

### Overproduction and purification of H-NS_C21S_

*E. coli* BL21 (DE3) *hns kan/frt* pLysE cells transformed with plasmid pRD41 were grown at 37 °C to an optical density of 0.4, and induced using IPTG (500 μM). Two hours after induction cells were collected and resuspended in buffer A (100 mM NH_4_Cl, 20 mM Tris pH 7.2, 10% glycerol, 10 mM β-mercaptoethanol (Sigma-Aldrich), 3 mM benzamidine). The cells were lysed by sonication and the lysate was cleared by centrifugation at 37000 rpm for 0.5 hours at 4 °C. The supernatant was loaded onto a P11 column pre-equilibrated with the same buffer. A 100 mM-1 M NH_4_Cl gradient was applied and the protein eluted at 280 mM NH_4_Cl. The buffer of the pooled peak fractions was replaced by buffer B (identical to buffer A, but containing 130 mM NaCl instead of NH_4_Cl) by overnight dialysis. The dialysate was loaded onto a pre-equilibrated heparin column (GE Healthcare). A 130 mM - 1 M NaCl gradient was applied and the protein eluted at 280 mM NaCl. The pooled peak fractions were again dialyzed against buffer B. Finally, the dialysate was concentrated using a 1 ml Resource-Q column and block elution using buffer B containing 300 mM NaCl (instead of 130 mM). The purity of the protein was verified on an SDS-PAGE gel. The protein concentration was determined using a Bicinchoninic Acid assay (Pierce BCA protein assay kit, Thermo Scientific).

### AFM imaging in air

Double-stranded DNA (dsDNA), diluted in TM buffer (20 mM Tris pH 7.0, 20 mM MgCl_2_), was deposited onto freshly cleaved mica and left to incubate for 20 min at room temperature^13,27^. Next, samples were rinsed with Milli-Q water and dried in a weak flow of nitrogen gas. AFM imaging was carried out on a Bioscope Catalyst™ system (Bruker, Leiderdorp, Netherlands) in the Peak Force Tapping^TM^ mode. We used SiN cantilevers (OMCL-RC800PSA, Olympus, Japan) with a nominal spring constant of 0.050 N/m. Scans were collected at a scan line frequency of 0.25 kHz at 512 × 512 pixel resolution.

### AFM imaging and force spectroscopy in liquid

First, 50 μl of thiol-labelled DNA (1.5 ng/μl) diluted in TNM buffer (10 mM Tris pH 7.0, 50 mM NaCl, 10 mM MgCl_2_) was deposited onto a gold coated glass surface (10 × 10 × 1 mm, 20 nm thick gold layer, Ssens, The Netherlands), and incubated for 10 min at room temperature. Samples were then rinsed three times with TNM buffer. For the DNA-H-NS experiments, 50 μl of 2μM H-NS_C21S_ (diluted in TNM buffer) was pipetted on the gold coated glass surface and incubated for 10 min and then rinsed three times with 1 ml TNM buffer. Next, after the removal of excess liquid, 100 μl TNM buffer was added to the sample. In the case of DNA-H-NS-DNA experiments, 2 μl of unlabeled DNA (108 ng/μl) was mixed into the sample, followed by 50 μl of 2 μM H-NS_C21S_ and incubation for 10 min. After rinsing three times with 1 ml TNM buffer, and removal of excess liquid, 100 μl TNM buffer was added to the sample and measurements were performed. Experiments without H-NS were performed as a control.

All measurements were performed in TNM buffer. The DNA, DNA-H-NS, or DNA-H-NS-DNA complexes were imaged at a resolution of 4 nm/pixel with a scan rate of 0.5 Hz in Peak Force Tapping^TM^ mode. Using these images, the position of each point for collecting pulling data was determined. Force spectroscopy: By applying a force of 5 nN as trigger threshold, individual DNA duplexes were picked up using the SiN cantilever (Olympus OMCL-RC800PSA) with a spring constant of ~0.1 N/m. The spring constant of each lever was precisely determined for each experiment by using the thermal tuning method^28^. The DNA molecule (or protein-DNA complex) was stretched between gold surface and tip upon retraction. The resulting force was measured through the deflection of the cantilever using optical lever detection. 5 to 10 approach/retract cycles were performed at each XY position. XY positions are spaced at least 25 nm apart to avoid pulling the same DNA molecule, yielding 500 to 1000 curves in a 2 μm by 2 μm area. The forward as well as the backward velocity during force spectroscopy measurements was set at 400 nm/sec, and 1024 data points for each F-D curve were recorded.

### Data analysis

Custom made programs, written in Labview^TM^, were used for analysis of force-distance (F-D) curves of pulling events. These curves recorded the force applied on the AFM tip as it approaches and retracts from the surface. The data analysis included curve screening, cantilever bending correction, curve alignment and Worm-Like Chain model fitting (equation 1) of each pulling event^29^.

1 $$F(z)=(\frac{{k}_{B}T}{{L}_{p}})\,(\frac{z}{{L}_{c}}-0.25+0.25{(1-\frac{z}{{L}_{c}})}^{-2})$$

Worm-like chain fitting formula, *K*_*B*_, *T*, *L*_*p*_ and *L*_*c*_ are Boltzmann’s constant, temperature, persistence and contour lengths of the DNA.

In short, the curves were translated along the x-axis to facilitate the superposition of F-D curves and to remove unspecific adhesion events at the beginning of the curve. Next the baseline and linear regions of the curve were removed and the contribution from the bending of the cantilever was subtracted. Finally, the corrected F-D curves of each measurement were compared with typical F-D curves of bare DNA. The pulling curves of bridged DNA-H-NS_C21S_-DNA complexes exhibit multiple breaking point events during the process of stretching. The force on the cantilever increased gradually and dropped repeatedly upon rupture of protein bridges between or in the DNA fragments. A custom-written program in Matlab^TM^ was used for analysing the force required for rupture of protein bridges. All force jumps below 25 pN were considered noise, and were not taken into account when analysing rupture forces generated by H-NS bridging.

The distance between ruptures was plotted out as a function of the probability of occurrence and then fit using an exponential decay function (equation 2).

2 $${P}_{Occurrence}={y}_{0}+A{e}^{-rx}$$

Exponential decay fitting formula, where x is the distance between rupture events, Y_0_ + A are initial starting values. r is the rate of decay.
